# Comparison between the Right and Left Distal Radial Access for Patients Undergoing Coronary Procedures: A Propensity Score Matching Analysis

**DOI:** 10.1155/2022/7932114

**Published:** 2022-07-21

**Authors:** Kristian Rivera, Diego Fernández-Rodríguez, Juan Casanova-Sandoval, Ignacio Barriuso, Marta Zielonka, Nuria Pueyo-Balsells, Immaculada Calaf Valls, Fernando Worner

**Affiliations:** ^1^University Hospital Arnau de Vilanova, Lleida, Spain; ^2^IRBLLeida (Institut de Recerca Biomèdica de Lleida), Lleida, Spain

## Abstract

**Introduction:**

Distal radial access for coronary procedures decreases hemostasis time, prevents radial occlusion, and improves patient comfort compared to conventional transradial access. Initially described for left distal radial access (lDRA), the right distal radial access (rDRA) is feasible. However, there are no comparative studies to date. This study aimed to evaluate the impact of the access site on vascular access and procedural performance.

**Methods:**

From August 2020 to October 2021, coronary procedures performed through distal radial access were prospectively recorded. After propensity score matching, the rDRA and lDRA were compared. The primary endpoint was the proportion of approach success. The secondary endpoints included access time, coronary procedural success, radial spasm, exposition to ionizing radiation, patient comfort, and vascular access-related complications.

**Results:**

From a total of 385 procedures in 382 patients, after a propensity score matching, 182 procedures were compared between the rDRA and lDRA. There were no differences in the baseline characteristics between the groups. Compared to the lDRA, the rDRA presented similar approach success (96.7% vs. 96.7%, *p*=1.0), less access time (39 (25–60) sec vs. 50 (29–90) sec, *p*=0.018), comparable coronary procedural success after sheath placement (100% vs. 100%, *p*=1.000), and not statistically significant radial spasm (2.19% vs. 6.59%, *p*=0.148). No differences in dose-area product (32 (20–56.2) Gy.m2 vs. 32.3 (19.4–46.3) Gy.m2; *p*=0.472) and fluoroscopy time (4.4 (2.5–9.1) min vs. 4.3 (2.4–7.5) min, *p*=0.251) were detected between the groups. No vascular access-related complications were observed in any group.

**Conclusions:**

The rDRA, compared to the lDRA, had the same proportion of approach success and procedural performance, with a slight reduction in access time for patients undergoing coronary procedures.

## 1. Introduction

Since the introduction and worldwide spread of the concept by Babunashvili and Kiemeneij, respectively, several studies have established the feasibility and safety of distal radial access (DRA) in the anatomical snuffbox for coronary procedures in addition to the reductions in hemostasis time, site puncture complications, and radial artery occlusion (RAO) compared with the conventional transradial access (TRA) [1, 2]. Furthermore, as the number of procedures has progressively increased, initial experiences with right distal radial access (rDRA) have been reported [3] despite the fact that the technique was first promoted using the left distal radial access (lDRA). To date, there are several clinical trials comparing the right versus left access with similar scenarios, such as the conventional TRA [4–8]. However, to our best knowledge, no comparative studies between the rDRA and lDRA are available for operators who want to adopt the distal radial approach.

Therefore, this study aimed to evaluate the impact of the access site on vascular access and procedural performance among patients undergoing diagnostic and/or therapeutic coronary procedures using the DRA.

## 2. Materials and Methods

### 2.1. Population and Study Design

Between August 2020 and October 2021, diagnostic and/or interventional coronary procedures performed between three expert operators using the DRA in a single center were prospectively included. The baseline clinical characteristics, preprocedural and vascular access characteristics, angiographic and procedural characteristics, and endpoints were collected and entered into a specific computerized database.

The study was conducted according to the principles of Helsinki Declaration and in compliance with current ethical and legal regulations and approved by the ethical committee (CEIC-2570). All patients gave written informed consent before coronary catheterization.

### 2.2. Inclusion and Exclusion Criteria

#### 2.2.1. Inclusion Criteria

1. Indication for invasive diagnostic and/or interventional coronary procedures.
2. Patients ≥18 years.
3. Normal Barbeau's test.

#### 2.2.2. Exclusion Criteria

1. Presence of a brachial arteriovenous fistula in the upper extremities.
2. Previous coronary artery bypass graft surgery.
3. Procedure performed during the learning curve.
4. Diameter of the distal radial artery <2.0 mm.
5. History of iodinated contrast allergy preventing the administration of premedication.
6. Women who were suspected to be pregnant.
7. Inclusion in other clinical trials or registries.

### 2.3. Endpoint Definitions

The primary endpoints was the DRA success. DRA success occurs when an introducer sheath can be properly placed through the punctured artery [9, 10].

The secondary endpoints were the time required for sheath insertion and the total procedure time, rate of coronary procedural success, development of radial spasm, exposition to ionizing radiation, patient comfort, and vascular complications related to access [10].

Access time was defined as the period from when the anesthesia needle contacts the skin to when the introducer sheath has been properly placed [9].

Radial artery spasm was defined based on a questionnaire addressing the following five signs: persistent forearm pain, pain response to catheter manipulation, pain response to introducer withdrawal, and difficulty in catheter manipulation after being “trapped” by the radial artery with considerable resistance on withdrawal of the introducer. Radial spasm was considered when at least two of the five signs were present or just one was present after the administration of a second dose of the spasmolytic agent, depending on the operator [11].

Exposure to ionizing radiation was evaluated using the dose-area product (DAP) in Gy.m2 and fluoroscopy time (min).

Patient comfort was evaluated using the visual analog scale (VAS) for pain related to the puncture site and hemostatic compression. A score of three or less for pain was defined as mild [2, 10].

The vascular complications related to access included radial artery occlusion, significant local hematoma, arterial dissection, pseudoaneurysms, and arteriovenous fistula [10].

Radial artery occlusion was defined as the absence of flow on Doppler color ultrasound (US) [10] after hemostasia device removal.

Forearm hematoma related to access was defined according to the recently modified EASY (Early Discharge After Transradial Stenting of Coronary Arteries Study) classification: Ia, distal to the styloid process of the radius; Ib, up to 5 cm proximal to the styloid process; II, up to 10 cm proximal to the styloid process; III, forearm; and IV, arm above the elbow [12].

### 2.4. Procedural Issues

All the procedures were performed under US guidance, using the rDRA or lDRA at the discretion of the interventional cardiologist.

To minimize arterial spasm, sublingual diazepam (10 mg) was administered 30 minutes before the administration of subcutaneous local anesthesia. A previous ultrasound (US) evaluation of the radial artery from the puncture site (DRA) to the brachial artery and a nonpathological Barbeau's test were mandatory before attempting the DRA.

The US-guided access technique has been described previously [2, 13]. Briefly, we scanned the distal radial artery with a transducer L25 × ((6–13 MHz) (FUJIFILM Sonosite, Bothell, WA)). Following a set order from the first dorsal web space to the anatomical snuffbox, the entire course of the radial artery to the brachial artery was evaluated to assess the tortuosity and artery size. Once the puncture site was chosen, in case of using the rDRA, the right hand was placed on the ipsilateral side of the patient in a natural position, flexing the thumb with slight ulnar deviation of the wrist. For the lDRA, the patient's left hand was moved as far as possible towards the right groin in a pronated position, flexing the thumb with slight ulnar deviation of the wrist. Then, 5 to 8 mL of subcutaneous mepivacaine 2% were injected. An US-guided puncture in the axial plane with a 21-gauge micropuncture needle was then performed using a single-wall technique. After US or fluoroscopy confirmation of the mini guidewire in the correct position, a 5F or 6F sheath was inserted (Prelude Ideal Hydrophilic Introducer Kit; Merit Medical Systems, South Jordan, Utah). Then, an intraarterial bolus with 2 mg of verapamil and 50 IU/kg of unfractionated heparin was administrated [14]. During the interventional procedures, unfractionated heparin was administered to complete 100 IU/kg and additional doses were administered to maintain the ACT between 250 and 300 sec [15]. The radial glide sheath was removed immediately after the procedure, and hemostasis was obtained by compression for 1–4 h with conventional compressive dressings. Once the compression time was over, we removed the wrapped gauze plug by loosening the elastic bandage, verifying the absence of bleeding at the puncture site. If the bleeding persisted, the gauze plug was replaced and maintained for one extra hour. A final US Doppler assessment of the radial artery was performed to confirm the vessel patency and detection of vascular access-related complications.

### 2.5. Statistical Analysis

Since the procedure (rDRA or lDRA) was not based on randomization, a propensity score matching (PSM) was performed to control for potential bias. The propensity scores were calculated using a logistic regression model with the access route as the dependent variable. Variables prior to the choice of the access route and type of coronary procedure were selected as independent variables and included age, gender, body mass index, hypertension, dyslipidemia, diabetes mellitus, smoking habit, previous myocardial infarction, previous stroke, previous heart failure, glomerular filtration rate before the procedure, left ventricular ejection fraction before the procedure, atrial fibrillation, type of anticoagulation therapy, previous coronary angiography, previous percutaneous coronary intervention, type of coronary angiography indication, outpatient coronary procedures, distal radial artery size, distal radial artery depth, and the type of coronary procedure. For the PSM, 1 : 1 protocol without replacement was used and the caliper was set at 0.1. A total of 91 pairs were matched. The predictive power of the model was 0.86 (95% confidence interval (CI), 0.84–0.87; *p* < 0.001) and the model was well-calibrated (Hosmer–Lemeshow; *p*=0.87). The data were analyzed according to the intention-to-treat principle (before potential crossover). The standardized differences and distribution of the propensity score are available in the section Supplementary Material. Categorical variables are expressed as count (percentage) and were compared using the chi-square test. Continuous variables are explored for normal distribution using the Kolmogorov–Smirnov test. Normally distributed variables are expressed as mean (standard deviation) and nonnormally distributed variables are expressed as median (interquartile range) and were compared using the t-student or U Mann–Whitney tests appropriately. All statistical analyses were performed using the SPSS Statistics 20.0 software (SPSS Inc., Chicago, IL, USA) and a 2-sided *p* < 0.05 was considered significant.

## 3. Results

**Figure 1:**
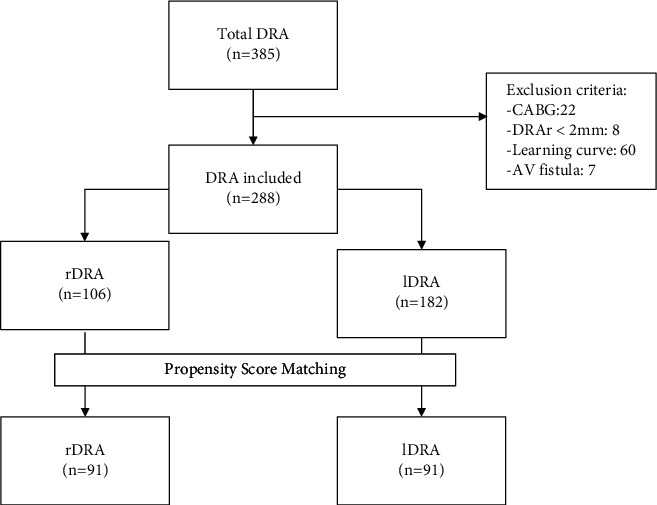
Study flowchart. DRA, distal radial access; CABG, coronary artery bypass graft; DRAr, distal radial artery; AV, arteriovenous; rDRA, right distal radial access; lDRA, left distal radial access.

A total of 385 procedures in 382 patients were performed between August 2020 and October 2021. After the PSM, 182 procedures were finally compared between the groups (rDRA vs. lDRA), and the results are as shown in Tables 1–4. The global analysis is available in the section Supplementary Material. The study flowchart is depicted in Figure 1.

### 3.1. Baseline Clinical Characteristics

The baseline clinical characteristics are shown in Table 1. There were no differences in the two groups.

### 3.2. Preprocedural and Vascular Access Characteristics

Data regarding preprocedural and vascular access characteristics are shown in Table 2. The proximal radial artery size (2.6 mm (0.6) vs. 2.9 mm (0.8); *p*=0.009) and the subsequent use of a 6F introducer sheath (73.6% vs. 59.3%; *p*=0.041) were superior in the lDRA group than in the rDRA group. No differences in clinical presentation, arterial pulse strength, distal artery size and depth, and postprocedural US radial evaluation were detected between the compared groups.

### 3.3. Angiographic and Procedural Characteristics

The angiographic and procedural characteristics are presented in Table 3. Only the extension of the coronary artery disease was higher in the rDRA group compared with the lDRA group (*p*=0.042). Once the sheath was inserted into the distal radial artery, all coronary procedures could be performed in both groups with no differences in the interventional or diagnostic procedures, use of specific techniques, procedure complexity, volume of contrast, and heparin dose between the groups.

### 3.4. Endpoints

Table 4 shows the comparative data of the endpoints. Compared to the lDRA, the rDRA presented similar approach success (96.7% vs. 96.7%; *p*=1.0). Less access time (39 sec (25–60) vs. 50 sec (29–90); *p*=0.035) was observed in the rDRA group than in the lDRA group. No differences in procedural time, radial artery spasm, and exposition to ionizing radiation were detected between the compared groups. The patient comfort during vascular access and hemostasis were high with no differences between the groups. Furthermore, after PSM, no vascular access-related complications were detected in any of the compared groups, including significant bleeding, forearm radial artery occlusion, arterial dissection, or significant hematoma related to the access.

## 4. Discussion

The main findings of this study were as follows: (a) the approach success was high with equivalent results between the rDRA and lDRA groups, (b) the access time was slightly lower in the rDRA group than in the lDRA group, and (c) coronary procedural success, radial spasm, ionizing radiation exposure, patient comfort, and vascular access-related complications were comparable between the rDRA and lDRA groups.

### 4.1. Equivalence of the Right and the Left Distal Radial Access

Eleven years after its initial description [1], the use of DRA was not yet widespread as expected [2, 4, 10, 12]. Despite the feasibility of the technique by the right access, to date, specific comparisons between the rDRA and the lDRA were not available for operators who prefer the right approach to perform coronary procedures, and this fact could limit the expansion of the technique.

Our results show that the rDRA and lDRA are equivalent with a high proportion of success, with a slight advantage in the access time for the rDRA compared to the lDRA (39 sec (25–60) vs. 50 sec (29–90); *p*=0.035). No differences in coronary procedural success, procedural time, radial spasm, exposition to ionizing radiation, or vascular access-related complications were detected, and the patient comfort for both approaches was similarly high in the two compared groups, in line with previous reports [2–4, 10, 12]. Furthermore, the spectrum of procedures performed using the DRA was very broad, showing the safety and feasibility of both distal approaches, even when performing intracoronary diagnostic imaging or complex percutaneous coronary interventions (such as rotational atherectomy and intracoronary lithotripsy) in multiple scenarios (such as bifurcations, left main artery, and chronic total occlusion).

Based on these results, we consider that both approaches are equivalent and operators could use the rDRA or the lDRA according to their own preferences or the specific characteristics of each patient. Below we detail some procedural aspects related with the procedure in our protocol.

### 4.2. Preprocedural Ultrasound Assessment and Distal Radial Access Technique

US-guided puncture offers advantages, such as the assessment of anatomical landmarks and accurate location of the puncture site [13], and improves patient selection [16, 17]. In our protocol, with the mandatory evaluation of the arteries by US prior to the procedure, only patients with the highest probability of success for DRA were selected, excluding patients with small-caliber arteries that are associated with more access failure. Consequently, the percentage of access success was very high (96.7%) in both groups in contrast to other studies that did not routinely use US and whose success rate was significantly lower [10, 12].

Furthermore, US, in addition to simplifying arterial puncture, could shorten the learning curve of the technique. The learning curves were variable depending on the type of vascular access. Nevertheless, a success rate above 95% can be considered a sign of overcoming the learning curve [18–20]. In our CathLab, after the first 20 cases per operator, the learning curve was achieved, observing that success exceeded the 95% threshold [21].

Also, relevant is the election of the puncture kit. The distal part of the radial artery presents a tortuous course as it crosses the snuffbox, which entails resistance when inserting the guidewire and the introducer sheath, promoting its collapse [9]. Thus, the employment of mini guidewires with floppy tip and stiff body and thin-walled hydrophilic sheaths with a suitable profile of sufficient rigidity, such as those provided by the puncture kit used in our CathLab [22], would allow the smooth insertion of the sheath while preventing its kinking.

Therefore, the selection of the appropriate material, as well as the preprocedural US assessment of potential candidates and the US-guided puncture, could facilitate success in DRA.

### 4.3. Limitations

First, this study was a single-center study, which could limit the extrapolation of the results to other populations. However, this is the first study comparing the right and left approaches for the DRA. Second, the nonrandomized nature of the study could affect the results. Nevertheless, PSM analysis probably minimizes potential biases derived from assignment to the rDRA or lDRA using the interventional cardiologist criteria. Third, the limited number of patients evaluated could underestimate the development of vascular access-related complications. Nevertheless, US-guided puncture was associated with reductions in vascular complications. Finally, US Doppler follow-up was not performed at 30 days; therefore, the benefits found in our study regarding radial artery patency are not available at midterm; nevertheless, at 5–12 months follow-up nonsignificant clinical complications were detected.

## 5. Conclusions

The performance of DRA procedures by right access was associated with equivalent approach success and procedural performance with a slight reduction in the access time compared to that of DRA with left access. Further studies are required to determine the most useful scenarios for rDRA in patients undergoing invasive coronary procedures.

## Figures and Tables

**Table 1:** Baseline clinical characteristics.

	Right distal radial access (*n* = 91)	Left distal radial access (*n* = 91)	*p* value
Age, (years), mean (SD)	67.9 (11.3)	69.05 (11.8)	0.501
Female gender, *n* (%)	30 (33.0%)	33 (36.3%)	0.640
BMI, (kg/m2), mean (SD)	27.35 (4.8)	27.03 (4.28)	0.634
Hypertension, *n* (%)	63 (69.2%)	66 (72.5%)	0.625
Dyslipidemia, *n* (%)	50 (54.9%)	47 (51.6)	0.656
Diabetes mellitus, *n* (%)	29 (31.9%)	29 (31.9%)	1.0
Smoking habit			0.114
Nonsmoker, *n* (%)	48 (52.7%)	62 (68.1%)	
Previous smoker, *n* (%)	28 (30.8%)	18 (19.8%)	
Current smoker, *n* (%)	15 (16.5%)	11 (12.1%)	
Family history of ischemic heart disease, *n* (%)	3 (3.3%)	6 (6.6%)	0.305
Previous MI, *n* (%)	17 (18.7%)	15 (16.5%)	0.353
Previous stroke, *n* (%)	4 (4.4%)	1 (1.1%)	0.174
Previous heart failure, *n* (%)	28 (30.8%)	30 (33.0%)	0.750
GFR (ml/minute/1.73 m^2^), mean (SD)	77.7 (16.2)	73.8 (17.2)	0.116
LVEF, mean (SD)	52 (17.1)	55 (15.8)	0.169
Atrial fibrillation, *n* (%)	13 (14.3%)	21 (23.1%)	0.128
OAT			0.697
Acenocoumarol, *n* (%)	11 (12.1%)	12 (13.2%)	
Dabigatran, *n* (%)	1 (1.1%)	1 (1.1%)	
Apixaban, *n* (%)	3 (3.3%)	6 (6.6%)	
Edoxaban, *n* (%)	0 (0%)	1 (1.1%)	

SD, standard deviation; BMI, body mass index; MI, myocardial infarction; GFR, glomerular filtration rate; LVEF, left ventricular ejection fraction; OAT, oral anticoagulation therapy.

**Table 2:** Preprocedural characteristics and vascular access characteristics.

	Right distal radial access (*n* = 91)	Left distal radial access (*n* = 91)	*p* value
Preprocedural characteristics			
Previous coronary angiography, *n* (%)	23 (25.3%)	18 (19.8%)	0.205
Previous PCI, *n* (%)	21 (23.1%)	18 (19.8%)	0.476
Coronary angiography indication			0.497
Chronic coronary syndrome, *n* (%)	19 (20.9%)	23 (25.3%)	
Acute coronary syndrome, *n* (%)	27 (29.7%)	19 (20.9%)	
Valvular heart disease, *n* (%)	21 (23.1%)	25 (27.5%)	
Myocardiopathy, *n* (%)	15 (16.5%)	18 (19.8%)	
Other, *n* (%)	9 (9.9%)	6 (6.6%)	
Outpatient coronary procedures, *n* (%)	48 (52.7%)	59 (64.8%)	0.098

Vascular access characteristics			
Arterial pulse strength scale			0.409
Absent	1 (1.1%)	0 (0%)	
Weak	12 (13.2%)	17 (18.7%)	
Normal	75 (82.4%)	73 (80.2%)	
Strong	3 (3.3%)	1 (1.1%)	
Distal radial artery size, mm (SD)	2.3 (0.2)	2.4 (0.3)	0.92
Proximal radial artery size, mm (SD)	2.6 (0.6)	2.9 (0.8)	**0.009**
Distal radial artery depth, mm (SD)	3.4 (0.1)	3.3 (0.1)	0.519
Introducer size			**0.041**
5 French, *n* (%)	37 (40.7%)	24 (26.4%)	
6 French, *n* (%)	54 (59.3%)	67 (73.6%)	
Postprocedural radial artery ultrasound evaluation, *n* (%)	91 (100%)	87 (95.6%)	0.076
Hemostasis time, (hour), mean, (SD)	2.6 (1.1)	2.8 (1.0)	0.350

PCI, percutaneous coronary intervention.

**Table 3:** Angiographic and procedural characteristics.

	Right distal radial access (*n* = 91)	Left distal radial access (*n* = 91)	*p* value
Angiographic characteristics			
LMCAD, *n* (%)	7 (7.7%)	3 (3.3%)	0.193
Number of diseased vessels			**0.042**
One vessel, *n* (%)	30 (33.0%)	49 (53.8%)	
Two vessels, *n* (%)	31 (34.1%)	20 (22.0%)	
Three vessels, *n* (%)	14 (15.4%)	10 (11.0%)	

Procedural characteristics			
Type of coronary procedures			0.745
Diagnostic, *n* (%)	64 (70.3%)	69 (75.8%)	
Interventional or combined, *n* (%)	27 (29.7%)	22 (24.2%)	
Specific techniques			0.341
FFR, *n* (%)	4 (4.4%)	5 (5.5%)	
OCT, *n* (%)	4 (4.4%)	0 (0%)	
IVUS, *n* (%)	1 (1.1%)	2 (2.2%)	
Catheter extender, *n* (%)	1 (1.1%)	2 (2.2%)	
Rotational atherectomy, *n* (%)	0 (0%)	1 (1.1%)	
Cutting balloon, *n* (%)	1 (1.1%)	3 (2.3%)	
Intracoronary lithotripsy, *n* (%)	1 (1.1%)	0 (0%)	
Thrombus aspiration, *n* (%)	4 (4.4%)	0 (0%)	
Special PCI procedures			0.296
Bifurcation, *n* (%)	2 (2.2%)	1 (1.1%)	
CTO, *n* (%)	1 (1.1%)	1 (1.1%)	
LMCAD, *n* (%)	1 (1.1%)	1 (1.1%)	
Volume of contrast, (mL), mean (SD)	82.1 (60.4)	73.5 (49.6)	0.294
Heparin dose, (IU), median (IQR)	4000 (3000–8000)	3500 (3000–6500)	0.349

LMCAD, left main coronary artery disease; FFR, fractional flow reserve; OCT, optical coherence tomography; IVUS, intravascular ultrasound; PCI, percutaneous coronary intervention; CTO, chronic total occlusion.

**Table 4:** Endpoints.

	Right distal radial access (*n* = 91)	Left distal radial access (*n* = 91)	*p* value
Primary endpoint			
DRA success	88 (96.7%)	88 (96.7%)	1.0

Secondary endpoints			
Access time, (sec), median (IQR)	39 (25–60)	50 (29–90)	**0.035**
Coronary procedural success after DRA	88 (100%)	88 (100%)	1.0
Procedural time, (min), median (IQR	27 (15–40)	25 (17–41)	0.360
Radial artery occlusion, *n* (%)	0 (0)	0 (0)	1.0
Radial artery spasm, *n* (%)	2 (2.2%)	6 (6.6%)	0.148
Hematoma, *n* (%)	0 (0)	0 (0)	1.0
DAP, (Gy.m2), median (IQR)	32 (20–56.2)	32 (19–46)	0.472
Fluoroscopy time, (min), median (IQR)	4.4 (2.5–9.1)	4.3 (2.4–7.5)	0.251
VAS patient comfort for access, mean (SD))	2.1 (0.3)	2.3 (0.7)	0.494
VAS patient comfort for hemostasia, mean (SD)	2.1 (0.2)	2.1 (0.3)	0.497

DRA, distal radial access, IQR, interquartile range; DAP, dose-area product; VAS, visual analog scale.

## Data Availability

The data used to support the findings of this study are included within the article.

## Supplementary Materials

Supplementary Materials Supplementary Figure 1: distribution of propensity scores. Supplementary Figure 2: standardized differences of propensity scores. Supplementary tables (global analysis of DRA before propensity score matching). Supplementary Table 1: baseline clinical characteristics. Supplementary Table 2: preprocedural characteristics and vascular access characteristics. Supplementary Table 3: angiographic and procedural characteristics. Supplementary Table 4: endpoints. Supplementary Table 5: vascular access-related complications.
