# Supplementary material for: Comparison between the Right and Left Distal Radial Access for Patients Undergoing Coronary Procedures: A Propensity Score Matching Analysis
Source: J Interv Cardiol. 2022 Jul 21;2022:7932114. doi: 10.1155/2022/7932114 (PMC9334045; doi:10.1155/2022/7932114)
Supplement: Supplementary Materials — Supplementary Figure 1: distribution of propensity scores. Supplementary Figure 2: standardized differences of propensity scores. Supplementary tables (global analysis of DRA before propensity score matching). Supplementary Table 1: baseline clinical characteristics. Supplementary Table 2: preprocedural characteristics and vascular access characteristics. Supplementary Table 3: angiographic and procedural characteristics. Supplementary Table 4: endpoints. Supplementary Table 5: vascular access-related complications. [file 7932114.f1.docx]

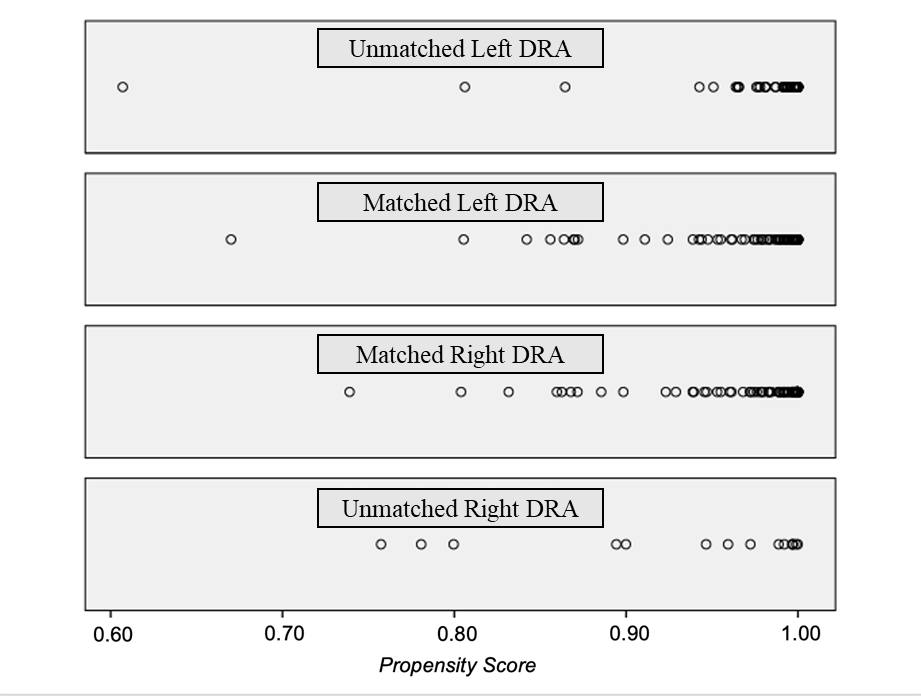


**Supplementary Figure 1. Distribution of propensity scores.**

**
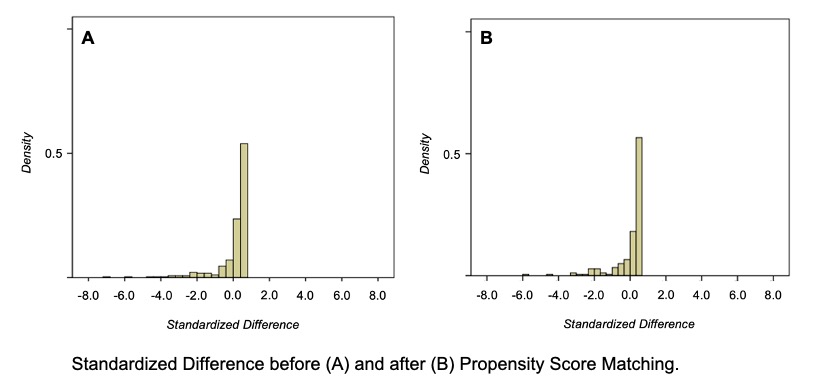
**

**Supplementary Figure 2. Standardized differences of propensity scores.**

**Global Analysis of DRA before propensity score matching.**

**Supplementary Table 1. Baseline clinical characteristics**

|  | **Right distal radial access**  **(n=106)** | **Left distal radial access**  **(n=182)** | **p-value** |
| --- | --- | --- | --- |
| **Age**, *(years), mean (SD)* | 68.63 (11.1) | 69.38 (11.5) | 0.588 |
| **Female gender**, *n (%)* | 38 (35.8%) | 62 (34.1%) | 0.759 |
| **BMI,** *(Kg/m2), mean (SD)* | 27.4 (4.7) | 27.39 (4.2) | 0.988 |
| **Hypertension**, *n (%)* | 75 (70.8%) | 136 (74.7%) | 0.463 |
| **Dyslipidemia**, *n (%)* | 57 (53.8%) | 99 (54.4%) | 0.745 |
| **Diabetes mellitus**, *n (%)* | 34 (32.1%) | 58 (31.9%) | 0.971 |
| **Smoking habit** |  |  | 0.520 |
| **Non smoker**, *n (%)* | 54.7%) | 113 (62.1%) |  |
| **Previous smoker**, *n (%)* | 31 (29.2%) | 43 (23.6%) |  |
| **Current smoker**, *n (%)* | 17 (16.0%) | 26 (14.3%) |  |
| **Family history of ischemic heart disease**, *n (%)* | 4 (3.8%) | 14 (7.7%) | 0.185 |
| **Previous MI**, *n (%)* | 18 (17.0%) | 34 (18.7%) | 0.194 |
| **Previous stroke**, *n (%)* | 4 (3.8%) | 6 (3.3%) | 0.838 |
| **Previous heart failure**, *n (%)* | 35 (33.0%) | 56 (30.8%) | 0.692 |
| **GFR** (ml/minute/1.73m^2^), *mean (SD)* | 77.42 (16.1) | 73.31 (18.6) | 0.580 |
| **LVEF,** *mean (SD)* | 52.19 (16.9) | 53.59 (16.4) | 0.488 |
| **Atrial fibrillation,** *n (%)* | 17 (16.0%) | 39 (21.4%) | 0.265 |
| **OAT** |  |  | 0.455 |
| **Acenocoumarol,** *n (%)* | 14 (13.2%) | 20 (11.0%) |  |
| **Dabigatran,** *n (%)* | 2 (1.9%) | 6 (3.3%) |  |
| **Apixaban,** *n (%)* | 3 (2.8%) | 11 (6.0%) |  |
| **Edoxaban,** *n (%)* | 0 (0%) | 3 (1.6%) |  |

SD, standard deviation; BMI, body mass index; MI, myocardial infarction; GFR, glomerular filtration rate; LVEF, left ventricular ejection fraction; OAT, oral anticoagulation therapy.

**Supplementary Table 2. Pre-procedural characteristics and Vascular access characteristics.**

|  | **Right distal radial access**  **(n=106)** | **Left distal radial access**  **(n=182)** | **p-value** |
| --- | --- | --- | --- |
| **Pre-procedural characteristics** |  |  |  |
| **Previous coronary arteriography,** *n (%)* | 23 (21.6%) | 43 (23.6%) | 0.718 |
| **Previous PCI**, *n (%)* | 21 (19.8%) | 40 (22.0%) | 0.702 |
| **Coronary angiography indication** |  |  | 0.334 |
| **Chronic coronary syndrome**, *n (%)* | 23 (21.7%) | 48 (26.4%) |  |
| **Acute coronary syndrome**, *n (%)* | 32 (17.6%) | 33 (18.1%) |  |
| **Valvular heart disease**, *n (%)* | 25 (23.6%) | 55 (30.2%) |  |
| **Myocardiopathy**, *n (%)* | 16 (15.1%) | 32 (17.6%) |  |
| **Other**, *n (%)* | 2 (1.9%) | 4 (2.2%) |  |
| **Outpatient coronary arteriography,** *n (%)* | 57 (53.8%) | 124 (68.1%) | **0.015** |
| **Vascular access characteristics** |  |  |  |
| **Arterial pulse strength scale** |  |  | 0.185 |
| **Absent** | 1 (0.9) | 0 (0) |  |
| **Weak** | 14 (13.2%) | 32 (17.6%) |  |
| **Normal** | 87 (82.1%) | 148 (81.3%) |  |
| **Strong** | 4 (3.8%) | 2 (1.1%) |  |
| **Distal radial artery size,** *mm (SD)* | 2.3 (0.2) | 2.4 (0.3) | **0.007** |
| **Proximal radial artery size,** *mm (SD)* | 2.63 (0.5) | 2.8 (0.7) | **0.009** |
| **Distal radial artery depth,** *mm (SD)* | 3.5 (1.1) | 3.5 (1.1) | 0.970 |
| **Introducer size** |  |  | **0.014** |
| **5 French,** *n (%)* | 44 (41.5%) | 50 (27.5%) |  |
| **6 French,** *n (%)* | 62 (58.5%) | 132 (72.5%) |  |
| **Post-procedural radial artery ultrasound evaluation,** *n (%)* | 106 (100%) | 175 (96.2%) | 0.110 |
| **Hemostasis time,** *(hour), mean, (SD)* | 2.7 (1.0) | 2.6 (1.1) | 0.461 |

PCI, percutaneous coronary intervention.

**Supplementary Table 3.** Angiographic and Procedural characteristics.

|  | **Right distal radial access**  **(n=106)** | **Left distal radial access**  **(n=182)** | **p-value** |
| --- | --- | --- | --- |
| **Angiographic characteristics** |  |  |  |
| **LMCAD**, *n (%)* | 7 (6.6%) | 6 (3.3%) | 0.192 |
| **Number of diseased vessels** |  |  | **0.036** |
| **One vessel**, *n (%)* | 36 (34.0%) | 94 (51.6%) |  |
| **Two vessels**, *n (%)* | 31 (29.2%) | 41 (22.5%) |  |
| **Three vessels**, *n (%)* | 19 (17.9%) | 23 (12.6%) |  |
| **Procedural characteristics** |  |  |  |
| **Type of coronary procedures** |  |  | 0.670 |
| **Diagnostics,** *n (%)* | 59 (64.8%) | 51 (56.0%) |  |
| **Interventional or combined,** *n (%)* | 32 (35.2%) | 40 (43.9%) |  |
| **Specific techniques** |  |  | 0.193 |
| **FFR,** *n (%)* | 4 (3.8%) | 10 (5.5%) |  |
| **OCT,** *n (%)* | 4 (3.8%) | 0 (0%) |  |
| **IVUS,** *n (%)* | 2 (1.9%) | 5 (2.7%) |  |
| **Catheter extender,** *n (%)* | 1 (0.9%) | 3 (1.6%) |  |
| **Rotational atherectomy,** *n (%)* | 0 (0%) | 2 (1.1%) |  |
| **Cutting balloon,** *n (%)* | 2 (1.9%) | 6 (3.3%) |  |
| **Intracoronary lithotripsy,** *n (%)* | 1 (0.9%) | 0 (0%) |  |
| **Thrombus aspiration,** *n (%)* | 5 (4.7%) | 1 (0.5%) |  |
| **Special PCI procedures** |  |  | 0.427 |
| **Bifurcation,** *n (%)* | 3 (2.8%) | 2 (1.1%) |  |
| **CTO,** *n (%)* | 1 (0.9%) | 3 (1.6%) |  |
| **LMAD,** *n (%)* | 1 (0.9%) | 1 (0.5%) |  |
| **Volume of contrast**, *(mL), mean (SD)* | 82.1 (58.1) | 74.1 (47.3) | 0.203 |
| **Heparin dose,** *(IU), median (IQR)* | 4000 (3000-7625) | 3000 (3000-6625) | 0.117 |

LMCAD, left main coronary artery disease; FFR, fractional flow reserve; OCT, optical coherence tomography; IVUS, intravascular ultrasound; PCI, percutaneous coronary intervention; CTO, chronic total occlusion.

**Supplementary Table 4.** Endpoints.

|  | **Right distal radial access**  **(n=106)** | **Left distal radial access**  **(n=182)** | **p-value** |
| --- | --- | --- | --- |
| **Primary Endpoint** |  |  |  |
| **DRA success** | 103 (97.2%) | 178 (97.8%) | 0.737 |
| **Secondary Endpoints** |  |  |  |
| **Access time,** (sec), *median (IQR)* | 36 (25-55) | 45 (30-90) | **0.018** |
| **Coronary procedural success after DRA** *n (%)* | 103 (100%) | 178 (100%) | 1.0 |
| **Procedural time***, (min), median (IQR* | 25 (16-42) | 25 (16-40) | 0.529 |
| **Radial artery spasm**, *n (%)* | 2 (1.9%) | 12 (6.6%) | 0.147 |
| **DAP***, (Gy.m2), median (IQR)* | 32 (19-58) | 28 (18-54) | 0.351 |
| **Fluoroscopy time***, (min), median (IQR)* | 4.4 (2.9-10) | 4.2 (2.3-7.5) | 0.114 |
| **VAS patient comfort for access,** mean  *(SD)* | 2.1 (0.3) | 2.2 (0.6) | 0.541 |
| **VAS patient comfort for hemostasia***, mean (SD)* | 2.1 (0.3) | 2.1 (0.5) | 0.490 |

DRA, distal radial access, IQR, inter-quartile range; DAP, dose-area product ; VAS, visual analogue scale.

**Suppementary Table 5.** Vascular access-related complications

|  | **Right distal radial access**  **(n=106)** | **Left distal radial access**  **(n=182)** | **p-value** |
| --- | --- | --- | --- |
| **Procedural complications** |  |  | 0.241 |
| **Bleeding,** *n (%)* | 1 (0.9%) | 0 (0%) |  |
| **Life threatening arrhythmias,** *n (%)* | 0 (0%) | 2 (1.1%) |  |
| **Radial artery occlusion,** *n (%)* | 0 (0%) | 2 (0.1%) |  |
| **Radial artery spasm**, *n (%)* | 2 (1.9%) | 12 (6.6%) | 0.147 |
| **Hematoma,** *n (%)* | 0 (0%) | 1 (0.5%) | 0.11 |
